# Editorial: Neuroimmunology in Africa

**DOI:** 10.3389/fimmu.2023.1201494

**Published:** 2023-05-02

**Authors:** Willias Masocha, Richard Idro, Roberto Furlan, Nouria Lakhdar-Ghazal

**Affiliations:** ^1^Department of Pharmacology and Therapeutics, College of Pharmacy, Kuwait University, Safat, Kuwait; ^2^College of Health Sciences, Makerere University, Kampala, Uganda; ^3^Institute of Experimental Neurology, Division of Neuroscience, San Raffaele Scientific Institute, Milan, Italy; ^4^Vita e Salute University, Milan, Italy; ^5^Department of Biology, Africa Center for Advanced Training in Neuroscience, Faculty of Sciences, Mohammed V University, Rabat, Morocco

**Keywords:** neuroimmunology, Africa, infectious diseases, noncommunicable diseases, immune cell, astrocytes, microglia, cytokine

## Neuroscience in Africa

The history of brain knowledge in our world dates back to the Pharaonic civilization of ancient Egypt. In the 17^th^ century BC, the Edwin Smith Papyrus gave the early reference to the brain by naming it “Marrow of the skull” (1). In this papyrus, the “Marrow of the skull” is recognized as composed of two hemispheres with circumvolutions (beginning of neuroanatomy) and wrapped in membranes; it is likely to spread a liquid (premises of immunology/neuroimmunology). From this period up to Galen, Egypt and then North Africa, were the center of knowledge about the human and animal brain (2, 3).

In the 1970s and 1980s, neuroscience research appeared sporadically in North and South Africa in the form of isolated publications from European-trained academics who joined universities in their home countries. Research was mostly clinical, developed on understanding diseases and developing treatments for neurological disorders such as epilepsy, motor disorders, but also infectious diseases: leprosy, tetanus, meningitis, encephalitis, which will later all come under the field of immunology and neuroimmunology. In some countries, research began to be structured around national associations or societies such as the Moroccan Association of Neuroscience (AMN; 1987), followed by the Southern African Society of Neurosciences (SANS; 1988), the Neuroscience Society of Nigeria (NSN; 1990) and Kenya Society for Neurosciences (KSN; 1992-1993). The Society of African Neuroscientists (SONA) was founded at the end of a scientific conference organized in 1993 in Kenya, federating the existing African Neuroscience Associations and Societies which were 4 at the time, and any individual researcher agreeing to be a member. The International Brain Research Organization (IBRO) has regularly supported all the activities of the SONA, which organizes a biannual congress. Other international organizations have also been involved in promoting SONA under various policies, the International Society for Neurochemistry (ISN) being a special example.

The need to take ownership of neuroscience training was felt and IBRO understood that it was necessary to sponsor neuroscience education in Africa for sustainable development. The first African school in neuroscience was organized in 2000 in South Africa. For more than 20 years now, IBRO has been generously supporting neuroscience schools and training workshops in more than 14 African countries. In addition to schools, two regular workshops were organized in conjunction with SONA conferences: Writing Papers workshop and Teaching Tools workshop. ISN has also contributed significantly to the funding of these activities. In 2015, IBRO created two regularly funded advanced neuroscience training centers (IBRO African Centers for Advanced Training in Neuroscience: ACATN): the first in Cape Town in 2015 and the second in Rabat in 2016. These centers were to organize two to three advanced training courses every year on value-added themes for Africa, with at least one regular school per center. The Cape Town center specialized in Computational neuroscience and the Rabat center on Basal Ganglia and Movement Disorders. In addition, the Rabat center hosted two neuroimmunology schools, in 2017 and 2019, supported also by the International Society of Neuroimmunology (ISNI) through the African School of Neuroimmunology. From these came the idea of this collection “Neuroimmunology in Africa”. This collection follows the path of previous collections focusing on neuroscience in Africa. A first collection appeared in Frontiers in Neuroanatomy on “Neuroscience in Africa” in 2019 (4), followed by another collection published as a Special edition of IBRO neuroscience reports titled “Neuroscience in Africa” in 2023 (5).

**Figure 1:**
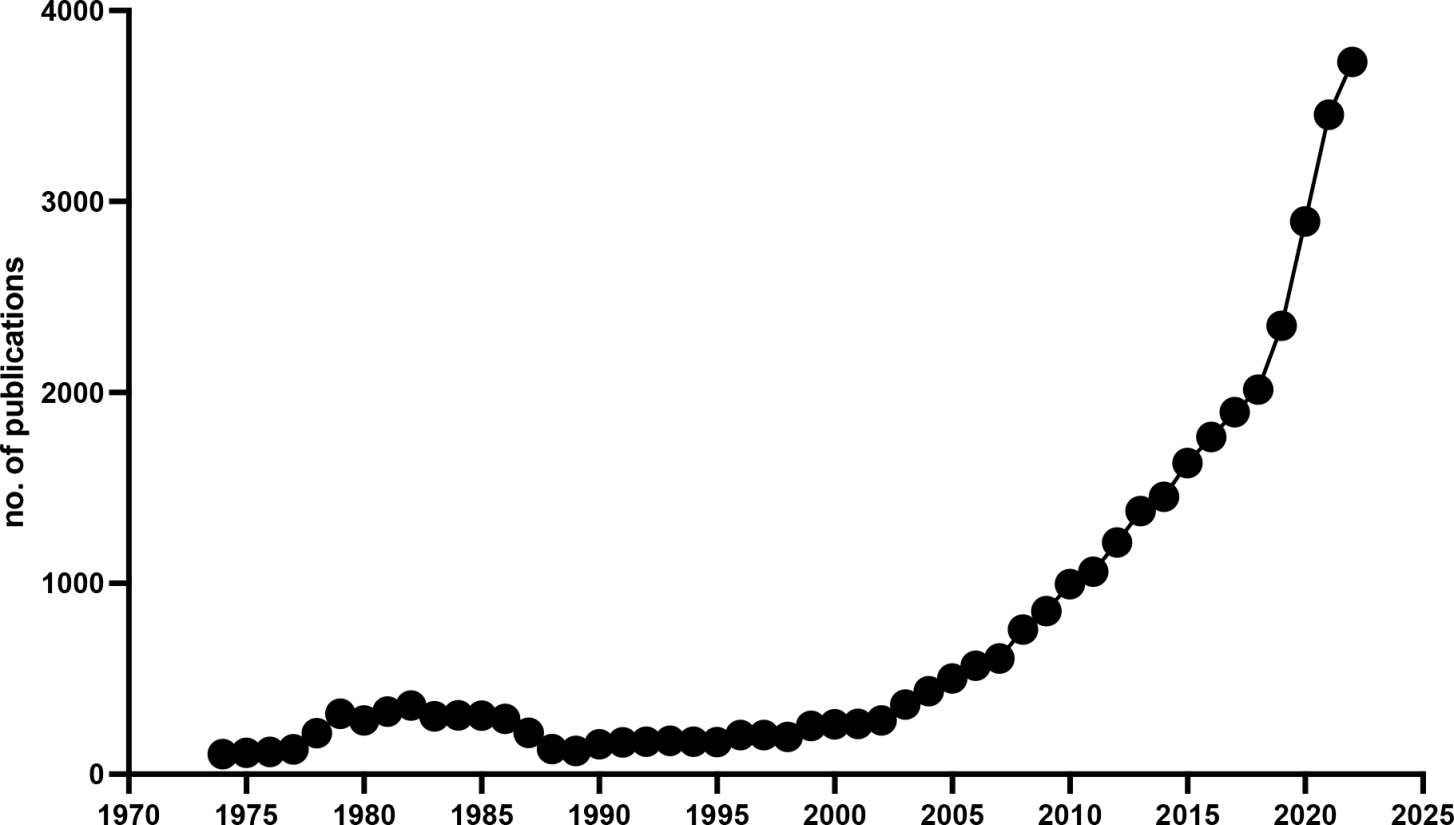
Number of neuroscience publications by authors with African institutional affiliations. Data extracted from Scopus using as keywords neuroscience, neurology, nervous system, brain, spinal cord, and African institutional affiliations.

The momentum that neuroscience in Africa has gained, boosted by these investments in education, can be appreciated in **Figure 1**, showing that the number of publications in this area co-authored by researchers with African institutional affiliations has been almost doubling every five years, starting from 1995.

This impressive growth was especially driven by nations like Algeria, Cameroon, Egypt, Ethiopia, Ghana, Kenya, Morocco, Nigeria, South Africa, although a steady increase, with a similar slope, can be appreciated also in nations with a more limited output in terms of neuroscience publications.

## Overview of the collection

The 14 reviews and one original research article in this Research Topic focus on the neuroimmunology of endemic diseases or leading causes of disease burden in Africa and can be divided into four main categories – general neuroimmunology, infectious diseases, neurological consequences and biomarkers of infectious diseases, and non-communicable diseases.

### General neuroimmunology

Mapunda et al. review how the immune cells cross various barriers such as the blood-brain barrier (BBB) and blood-cerebrospinal fluid barrier and get into the brain, during multiple sclerosis (MS). The article also explores the influence of genetic and environmental factors on how immune cells enter the CNS during neuroinflammation, with special emphasis on Africa. The role of different T helper cells, such as Th1, Th17, GM-CSF-producing Th cells, and cytokines in neuroinflammation and neurodegeneration are covered by Krishnarajah and Becher. Olude et al. review astrocytes and microglia including their physiology, crosstalk between them and the role they play in health and disease, emphasizing the African perspective in the context of stressors such as malnutrition, developmental stress, and environmental pollutions.

### Infectious diseases

The total disease burden in Africa is still dominated by communicable diseases (6). Six reviews cover neuroinfections caused by viruses, bacteria, fungi, and parasites. Human immunodeficiency virus (HIV) infections disproportionally affect Africa. Meyer et al. describe the neuroimmunology of HIV CNS infection. CNS injury is caused by the virus, opportunistic infections, and local immune inflammatory reactions. Immune cells and cytokines from the periphery also cause CNS neuroinflammation. Klein reviews the neuropathogenesis of specific endemic mosquito-borne viruses (arboviruses) of the *Flaviviridae* family (such as West Nile virus and Zika virus) and *Togaviridae* family (such as chikungunya virus and Sindbis virus). Neurotropic arboviruses enter the CNS through retrograde transport of virus along axon microtubules of peripheral neurons, infection of olfactory sensory neurons or through the BBB. Scott and Nel describe rabies lyssavirus (RABV) endemic in Africa, that cause the fatal encephalitic disease rabies. Pathogenic RABV strains inhibit innate immune signaling, induce cellular apoptosis and use viral protein to facilitate retrograde axonal transport of the virus to the CNS. Idro et al. review parasites that infect the CNS such as *Plasmodium falciparum*, *Toxoplasma gondii*, *Trypanosoma brucei* spp., and *Taenia solium* species. The article explains the role of the immune system in neuroinvasion, control and neuropathogenesis of parasites. Mohamed et al. cover fungal CNS infections in Africa, with special emphasis on the neuroimmunology of cryptococcal meningitis, which is the leading cause of CNS fungal infections in humans. Barichello et al. describe bacterial meningitis in Africa, the common bacteria that cause it, how the bacteria get to the brain, interactions of the bacteria with neurons, and the role of microglia and cytokines play in the neuroinflammation associated with bacterial meningitis.

### Neurological consequences and biomarkers of infectious diseases

Ngarka et al. sum up the interplay between neuroinfections, the immune system and neurological disorders with a special emphasis on neurological diseases common in Africa as a sequelae of neuroinfections. Neurological disorders associated with HIV infection such as HIV-associated neurocognitive disorders, motor disorders, chronic headaches, and peripheral neuropathy are high in the sub-Saharan region because of high prevalence of HIV. The immune system deregulation in addition to the virus and antiretroviral drugs contribute to these neurological disorders. Infections such as toxoplasmosis, neurocysticercosis, onchocerciasis, malaria, bacterial meningitis, tuberculosis, and the immune reactions they elicit contribute to the high prevalence of epilepsy on the continent. Other neurological disorders attributable to neuroinfections and the neuroimmune response they trigger include sleep disorders, secondary headaches, dementia, motor neuron diseases. Ihunwo et al. explain how some viruses can get to the brain and affect neurogenesis. Zika virus can infect fetal brain and affect neural stem cells, neurogenesis, synaptogenesis, and cause cell death, with severe consequences such as microcephaly and decreased brain tissue. Severe acute respiratory syndrome coronavirus 2 can infect the olfactory bulb and travel to the CNS by retrograde axonal transport along olfactory sensory neurons, target neurons, astrocytes, and microglia and result in neurological symptoms observed in coronavirus disease 2019 (COVID-19) patients. Ndondo et al. review post-infectious autoimmunity in the CNS and peripheral nervous systems, pointing out the peculiarities in Africa. They cover the various conditions that occur after viral infections such as acute necrotizing encephalopathy, measles-associated encephalopathies, HIV neuroimmune disorders, and difficulties associated with classical post-infectious autoimmune disorders such as the Guillain-Barré syndrome in the context of HIV and other infections. NMDA-R encephalitis and myasthenia gravis, as the classic antibody-mediated disease, are also covered. Teunissen et al. summarize research in the use of biomarkers in tuberculous meningitis and pediatric HIV. They explain the possible diagnostic and prognostic values of some inflammatory molecules, such as cytokines and chemokines, and brain injury molecules, such as S100, neuron specific enolase and glial fibrillary acidic protein, when detected in the CSF. The only original research article in the theme by Bertran-Cobo et al., conducted in South Africa, found that myo-inositol, a marker for glial reactivity and inflammation, was elevated in children who are HIV-exposed and uninfected, which points to ongoing neuroinflammatory processes that may contribute to developmental risk in these children.

### Non-communicable diseases

The prevalence of non-communicable diseases is increasing on the African continent (6). Ballerini et al. review non-communicable neurological disorders and neuroinflammation, focusing on traumatic brain injury (TBI), stroke, and neurodegenerative diseases such as dementias because they represent a major cause of morbidity and mortality in Africa. Neuroinflammation, encompassing glial cell activation and cytokine secretion, is a major factor in the pathobiology of TBI and stroke. In Alzheimer’s disease, neuroinflammation is both a reaction against and a contribution to the neurodegenerative pathology.

## Conclusions

All these articles emphasize the importance of the subject of neuroimmunology in Africa, in some cases because of the peculiarities the continent has in terms of infectious diseases but also for its importance to healthcare including diagnosis, treatment and understanding the neurological disorders that occur as sequalae of infectious diseases as well as non-communicable neurological disorders. Various knowledge gaps are highlighted that necessitates further research in these various disorders. This research will not only benefit the African continent but the world at large in understanding the CNS, neuroimmunology and neuroinflammation. For example, trypan dyes developed by Paul Ehrlich in search of drugs to kill African trypanosomes aided Edwin E. Goldmann to discover the BBB (7).

## Author contributions

All authors listed have made a substantial, direct, and intellectual contribution to the work, and approved it for publication.
